# Correction: Novel evidence that extracellular nucleotides and purinergic signaling induce innate immunity-mediated mobilization of hematopoietic stem/progenitor cells

**DOI:** 10.1038/s41375-019-0407-y

**Published:** 2019-03-08

**Authors:** Mateusz Adamiak, Kamila Bujko, Monika Cymer, Monika Plonka, Talita Glaser, Magda Kucia, Janina Ratajczak, Henning Ulrich, Ahmed Abdel-Latif, Mariusz Z. Ratajczak

**Affiliations:** 1 Stem Cell Institute at James Graham Brown Cancer Center, University of Louisville, Louisville, KY USA; 2 Department of Regenerative Medicine and Center for Preclinical Studies and Technology, Warsaw Medical University, Warsaw, Poland; 3 Department of Biochemistry, Institute of Chemistry, University of São Paulo, São Paulo, Brazil; 4 Division of Cardiovascular Medicine, Gill Heart Institute, University of Kentucky, Lexington, KY USA

**Keywords:** Haematopoietic stem cells, Cell signalling

**Correction to:** **Leukemia** **32**; 1920–1931 (2018);

10.1038/s41375-018-0122-0;

published online: 30 March 2018

Following the publication of this article, the authors noted that the following should be included in the Acknowledgements section: “MA is the recipient of a START scholarship (0785) from FNP”. The authors wish to apologise for any inconvenience caused.

